# Bone and the Unfolded Protein Response: In Sickness and in Health

**DOI:** 10.1007/s00223-023-01096-x

**Published:** 2023-05-27

**Authors:** Srividhya Iyer, Douglas J. Adams

**Affiliations:** Department of Orthopedics, University of Colorado Anschutz Medical Campus, 12800 E 19th Ave, Mailstop:8343, Aurora, CO 80045 USA

**Keywords:** Protein misfolding, Proteostasis, IRE1, PERK, ATF6, ER stress

## Abstract

Differentiation and optimal function of osteoblasts and osteoclasts are contingent on synthesis and maintenance of a healthy proteome. Impaired and/or altered secretory capacity of these skeletal cells is a primary driver of most skeletal diseases. The endoplasmic reticulum (ER) orchestrates the folding and maturation of membrane as well as secreted proteins at high rates within a calcium rich and oxidative organellar niche. Three ER membrane proteins monitor fidelity of protein processing in the ER and initiate an intricate signaling cascade known as the Unfolded Protein Response (UPR) to remediate accumulation of misfolded proteins in its lumen, a condition referred to as ER stress. The UPR aids in fine-tuning, expanding and/or modifying  the cellular proteome, especially in specialized secretory cells, to match everchanging physiologic cues and metabolic demands. Sustained activation of the UPR due to chronic ER stress, however, is known to hasten cell death and drive pathophysiology of several diseases. A growing body of evidence suggests that ER stress and an aberrant UPR may contribute to poor skeletal health and the development of osteoporosis. Small molecule therapeutics that target distinct components of the UPR may therefore have implications for developing novel treatment modalities relevant to the skeleton. This review summarizes the complexity of UPR actions in bone cells in the context of skeletal physiology and osteoporotic bone loss, and highlights the need for future mechanistic studies to develop novel UPR therapeutics that mitigate adverse skeletal outcomes.

## Introduction

The adult bone marrow stroma contains a heterogenous subset of tissue resident skeletal stem/progenitor cells (SSCs) that can self-renew and give rise to multiple lineages that comprise the skeleton (osteoblasts, cartilage, adipocytes, supporting stroma) [1–3]. Proliferation and differentiation of SSCs of mesenchymal origin into bone matrix-secreting osteoblasts is directed by the transcription factors Runx2 and Osterix (Osx1) by integrating signals from bone morphogenetic protein (BMP), fibroblast growth factor (FGF), Wnt, Notch, and Indian hedgehog signaling pathways [4]. Osteoblasts are short-lived and secrete multiple proteins (e.g., collagen, osteocalcin, alkaline phosphatase) that make up the bone matrix. The metamorphosis of SSCs into matrix-synthesizing cells occurs with expansion of the ribosome-laden endoplasmic reticulum (ER) early during differentiation [5], emphasizing the critical role of this organelle in the synthesis of secretory and membrane proteins. Indeed, mature osteoblasts feature a profuse rough ER [6–9] that is leveraged for correct processing of collagen and non-collagenous proteins which comprise the bone matrix. Some osteoblasts embed in the bone matrix and differentiate into osteocytes, which, unlike osteoblasts, are long-lived [10]. The osteocytes also secrete a variety of factors that control bone formation and erosion (resorption), mechanical adaptation, and mineral homeostasis. [10, 11]. As part of skeletal growth and remodeling, osteoclasts resorb cartilaginous anlagen and bone matrix by secreting enormous amounts of enzymes including collagenases, cathepsin K, metalloproteases, and other hydrolytic enzymes [12]. The transcription factor nuclear factor of activated T cells cytoplasmic 1 (NFATc1) orchestrates the differentiation of osteoclasts from macrophages upon stimulation by the cytokine receptor activator of NFκB ligand (RANKL) [12, 13]. Continued replacement of older, possibly damaged, bone that is resorbed by osteoclasts with an equivalent amount of new bone is essential for skeletal health and structural integrity. The differentiation and activity of osteoblasts and osteoclasts required for this skeletal ‘remodeling’ is reliant on the synthesis of a multitude of membrane and secreted proteins.

**Fig. 1:**
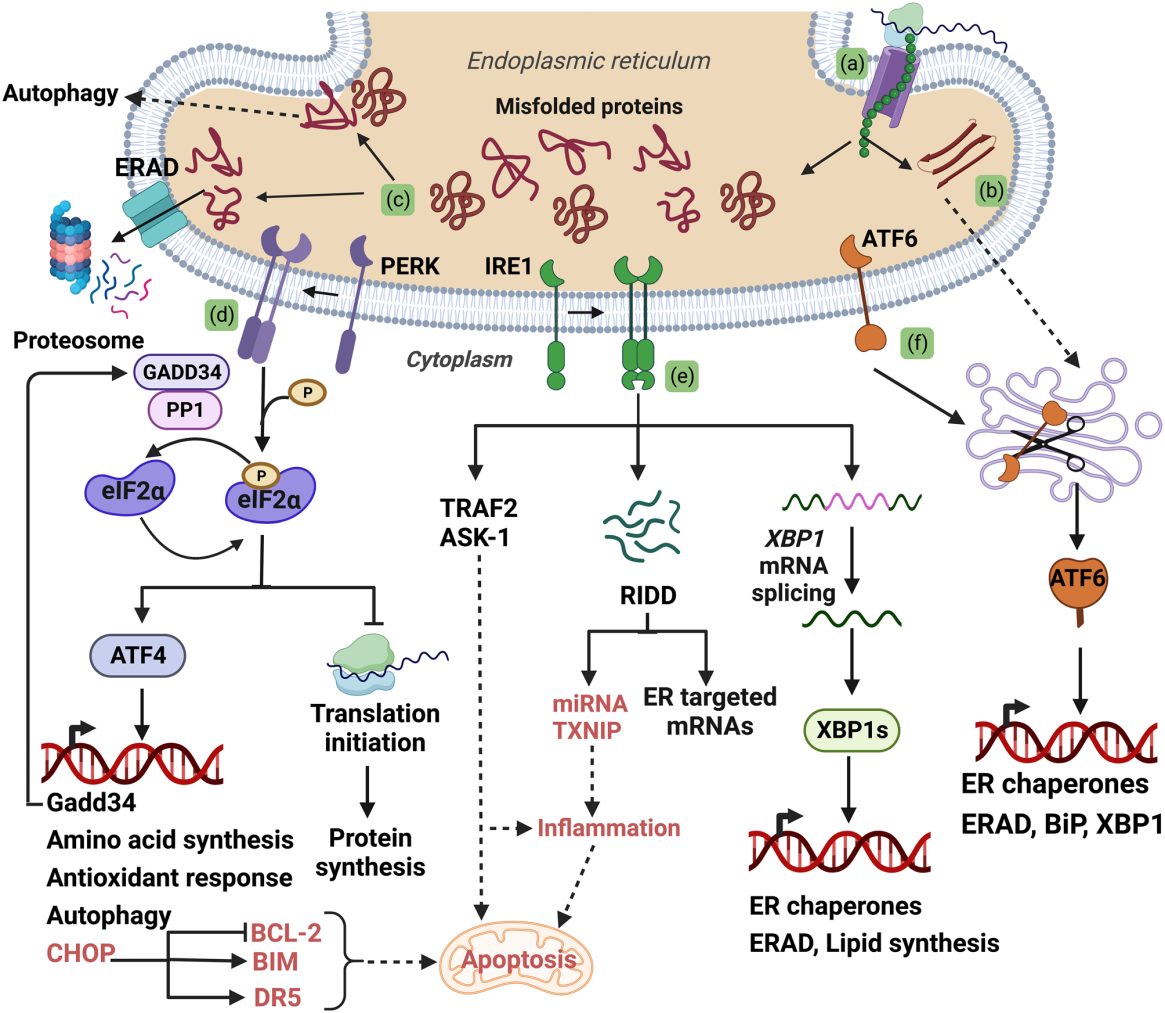
Surveillance of protein processing by the UPR. (a) Secretory and membrane proteins co-translationally enter the ER and are acted upon by ER chaperones such as BiP. (b) Proteins that attain a functional conformation are trafficked via the Golgi processes (c) Misfolded proteins are directed for either proteasomal or lysosomal degradation via ERAD or autophagy, respectively. (d, e, f) PERK, IRE1 and ATF6 monitor protein processing and initiate the UPR in response to accumulation of misfolded proteins in the ER stress. (d) Upon stress, PERK oligomerizes and gets activated by autophosphorylation. Active PERK phosphorylates eIF2α and attenuates protein synthesis leading to selective translation of ATF4. ATF4 stimulates of amino acid synthesis, antioxidant genes, autophagy, and expression of Gadd34 and CHOP. GADD34 restores protein synthesis by reversing eIF2α phosphorylation. If ER stress persists, ATF4/ CHOP signaling triggers apoptotic program. (e) The RNase activity of the active IRE1 dimer splices XBP1 mRNA in response to ER stress. Spliced XBP1 (XBP1s) encodes a transcription factor that augments expression of ER chaperones and ERAD components. Degradation of ER targeted mRNAs by IRE (RIDD) reduces protein processing loads. Hyperactivated IRE1 can associate with TRAF2 and ASK proteins to initiate JNK and caspase mediated inflammation and apoptotic programs. RIDD mediated decay of TXNIP miRNA can lead to sterile inflammation and apoptosis. (f) ATF6, proteolytically activated upon translocation to the Golgi processes, release a bZIP transcription factor that induces expression of BiP, XBP1 and genes involved in ER proteostasis. The apoptotic outputs are highlighted in red. Figure created with Biorender.com

Approximately, one-third of the eukaryotic proteome, comprising membrane and secretory proteins, is routed through the endoplasmic reticulum (ER). Folding of proteins, especially membrane receptors with multiple domains, requires exquisite spatial and temporal coordination [14]. The nascent polypeptides co-translationally enter the ER lumen where they acquire their native 3D conformation. The tubular network of the ER also serves as a site for lipid biosynthesis and an intercellular Ca^+2^ reservoir. Its proximity to the nucleus, mitochondria, Golgi processes and lysosomes render it a critical scaffold for intraorganellar communication [15]. The ER hosts a multitude of molecular chaperones, foldases, isomerases, and oxidoreductases with a distinctly oxidative milieu that favors protein folding [14, 16]. The ER resident enzymes (i) direct each nascently synthesized polypeptide into folding intermediates which minimizes their aggregation and misfolding (Fig. 1(a)) and (ii) mediate posttranslational modifications such as glycosylation and disulphide bond formation. Proteins that obtain their functional conformation are directed to the Golgi bodies for secretory processing (Fig. 1(b)). Precise structure is crucial for optimal protein function; therefore, protein folding and processing in the ER are subject to stringent quality control mechanisms.

Proteins that fail to be properly processed in the ER are recognized as misfolded and promptly directed for ER-associated degradation (ERAD) (Fig. 1(c)) [17, 18]. ERAD comprises chaperones, enzymes, and an ER membrane transport channel that ubiquitinate the misfolded client peptides as they exit from the ER into cytosol to aid their rapid degradation. Fragments of the ER, containing protein aggregates that are resistant to ERAD, can also be tagged with ER-specific membrane factors and directed for autophagy (known as ER-phagy) (Fig. 1(c)) [19]. Autophagy is a complex catabolic process that mediates removal of damaged organelles, misfolded proteins, and aggregated proteins within the cell [20].

Perturbations in pH, oxygen tension, cellular redox, deficits in glucose or energy , or increased demand for protein synthesis can disrupt homeostasis and protein processing within the ER [18, 21, 22]. Consequent accumulation of unfolded proteins in its lumen, commonly referred to as ER stress, activates an intricate signaling cascade in mammalian cells known as the Unfolded Protein Response (UPR) [23]. Depending on the intensity and duration of ER stress, the UPR enforces an adaptive or apoptotic cellular program [21, 23]. Here, we discuss how the UPR pathways aid maintenance of a healthy proteome in skeletal cells and its relevance in physiologic and pathologic settings. The literature on UPR biology is enormous. Select reviews have been referenced for background on the UPR, which comprise individual contributions.

### UPR Mediated Oversight of ER Homeostasis

Three ER transmembrane proteins known as inositol-requiring enzyme-1 (IRE1), activating transcription factor (ATF6), and protein kinase RNA (PKR)-like ER kinase (PERK) serve as ER stress sensors and initiate a sequalae of interconnected signaling pathways comprising the UPR [24]. The mammalian genome encodes two isoforms of IRE1 and ATF6 [25, 26]. IRE1α, ATF6α and ATFβ isoforms are ubiquitously expressed. IRE1β expression has been described in epithelial cells lining  the intestine and other mucosal surfaces, but not in the skeleton. The alpha isoforms of IRE1 and ATF6 have been studied extensively in the context of the UPR and ER stress, including in the skeleton, and are discussed further in this review.

In response to ER stress, activation of the UPR transducers can occur either via direct binding of unfolded proteins to their luminal domain, or misfolding-induced dissociation of the ER chaperone BiP/Grp78 (detailed mechanisms reviewed in [27]). To mitigate the protein processing overload in the ER, two distinct cellular responses are initiated as part of the UPR program- (i) the global suppression of protein synthesis and (ii) transcriptional upregulation of genes that augment folding capacity of the ER, antioxidant responses, and misfolded protein clearance by ERAD and autophagy. Failure to resolve ER stress by these adaptive measures leads to excessive/continued activation of the UPR sensors, which in turn leads to cell death. A brief description of the how signaling transduction by the UPR follows. For detailed reviews on mechanisms that underlie UPR activation please refer to reviews by Hetz and colleagues [21, 23, 24, 28].

#### Adaptive UPR

PERK is a type1 transmembrane protein with serine/threonine kinase activity in its cytosolic domain [21, 24, 28]. In response to ER stress, PERK oligomerizes and activates by autophosphorylation (Fig. 1(d)). Activated PERK represses global protein synthesis by phosphorylating the α subunit of eukaryotic initiation factor 2 (eIF2α) at serine 51, an event that presumably reduces protein processing loads in stressed cells. The eIF2αβγ heterotrimer complexes with guanosine-5'-triphosphate (GTP)and mediates initiation of translation [29, 30]. Phosphorylation of eIF2α limits the levels of active eIF2-GTP complex and attenuates global translation. Phosphorylated (p)-eIF2a also initiates an adaptive remodeling of the proteome via selective translation of mRNAs harboring short upstream open reading frames [21, 23, 24]. The transcription factor ATF4 is predominant among the genes upregulated [31]. ATF4 activates the expression of genes that regulate antioxidant response, autophagy, and amino acid metabolism [32]. The transience of p-eIF2α-induced translational arrest is ensured by ATF4-dependent upregulation of growth-arrest DNA damage-inducible protein (GADD34). GADD34 encodes the regulatory subunit of protein phosphatase 1 (PP1) that dephosphorylates p-eIF2α, which in turn restores protein synthesis and attenuates ATF4 translation [33]. In addition to PERK, three other kinases (general control non‐depressible protein 2 (GCN2), heme‐regulated eIF2α kinase (HRI) and PKR), can stimulate p-eIF2α mediated proteome remodeling, and collectively comprise the Integrated Stress Response (ISR) [21, 24]. The ISR kinases can be triggered by varied stimuli including nutrient deprivation, hypoxia, osmotic or heat shocks, viral infections, and oxidative stress.

IRE1 is the most evolutionarily conserved arm of the UPR [21, 24]. Akin to PERK, ER stress induces oligomerization and autophosphorylation of IRE1 (Fig. 1(e)). Once activated, the cytosolic endoribonuclease (RNAse) domain of IRE1 exerts three distinct signaling outputs [21, 24]. IRE1 mediates excision of 26 nucleotides from the mRNA encoding X-Box binding protein (XBP1). This non-canonical splicing shifts the open reading frame of mRNA during translation, generating a spliced variant of XBP1 mRNA (XBP1s). XBP1s encodes a basic leucine zipper (bZIP) transcription factor that regulates the expression of genes involved in protein translocation into the ER, its folding and trafficking to Golgi vesicles, ERAD, and lipid synthesis [34]. The RNAse activity of IRE1 also degrades select mRNA substrates that enter the ER in a cell type-dependent manner [35]. This process, termed Regulated IRE1-Dependent Decay (RIDD), is thought to alleviate the load of protein folding akin to p-eIF2α. IRE1 can also function as a signaling scaffold by associating with adaptor proteins such as TRAF2, apoptosis signal-regulating kinase 1 (ASK1) and Nck to mediate crosstalk with JUN N-terminal kinase (JNK), mitogen activated protein kinase (MAPK) and NFkB stress response pathways [21, 24].

ATF6 constitutes the third arm of the UPR [21, 24]. This type 2 transmembrane protein contains a bZIP transcription factor in its cytosolic domain. Upon ER stress, ATF6 translocates to the Golgi where it is cleaved by S1P and S2P proteases to release an active ATF6 transcription factor domain that localizes to the nucleus (Fig. 1(f)). ATF6 stimulates expression of XBP1, a subset of ER chaperones including BiP, and reinforces ERAD [18, 21]. At the protein level, ATF6 and XBP1s can form homo or heterodimers and induce expression of an overlapping but distinct set of genes that participate in protein processing and Golgi apparatus biogenesis to expand the secretory capacity of the cells [18, 21].

#### Terminal UPR

Failure to restore ER proteostasis by the ‘adaptive UPR’ program hastens cell death. If ER stress remains irremediable, sustained activation of the PERK and IRE1 axes can modulate several factors that participate in and/or engage pro-apoptotic outputs. Persistent activation of PERK can upregulate expression of the transcription factor CCAAT/enhancer-binding protein homologous protein (CHOP) downstream of ATF4 (Fig. 1(d)) [21, 24, 28]. CHOP inhibits expression of anti-apoptotic B-cell lymphoma-2 (BCL-2) protein. CHOP also upregulates pro-apoptotic Bcl-2 Interacting Mediator of cell death (BIM) protein as well as the death receptor 5 (DR5)/caspase-8 axis. Persistent GADD34 and CHOP activity increases oxidative stress and proteotoxicity within the cells, sensitizing them to apoptosis. Prolonged phosphorylation of IRE1 induces its assembly as a higher order oligomer that attenuates XBP1 splicing and favors RIDD activity [21]. This switch in IRE1 substrate preference limits the availability of ER chaperones and protein processing factors, and dampens a major pro-survival avenue. Unmitigated ER stress can also expand the cellular RIDD clientele to include miRNAs that repress thioredoxin-interacting protein (TXNIP) and caspase-2, thus initiating sterile inflammation and apoptosis (Fig. 1(e)) [21]. In some cellular systems, however, caspase-2 is dispensable for ER stress-mediated apoptosis [36]. Chronic ER stress also promotes assembly of IRE1/TRAF2 complexes that stimulate proapoptotic JNK signaling. In some studies, IRE1 has been shown to elicit mitochondrial apoptosis by complexing with BCL2 associated X (BAX) and BCL2 antagonist/killer 1 (BAK) via its cytosolic domain [37]. Notably, BAX-inhibitor 1 (BI-1) attenuates the proapoptotic activity of IRE1 by direct interaction, presumably by competing for a similar binding site. Unresolved ER stress and dysregulation of the UPR are extensively and causally implicated in the pathophysiology of obesity, diabetes, cancer, immune diseases and neurodegenerative disorders [21, 24].

#### Crosstalk within the UPR

Some level of connectivity exits between various outputs of the UPR to pace cellular responses to ER stress. For instance, in the adaptive phase of the UPR, PERK can stimulate ATF6 expression and processing to augment recovery from stress [38]. The unspliced XBP1 transcript can attenuate XBP1s and ATF6 levels, limiting the duration of the adaptive phase [39, 40]. Recent RNA-seq and ribosome profiling demonstrate that PERK can repress the cytoprotective genes induced by XBP1s and ATF6 [41]. PERK can also attenuate the adaptive IRE1/XBP1s response and initiate apoptosis via the phosphatase RNA polymerase II-associated protein 2 (RPAP2), which reverses IRE1 phosphorylation [42]. These observations support a model wherein the activities of various UPR outputs seamlessly integrate as the response to ER stress progresses, and coordinate a cell-survival versus apoptotic fate [43].

#### Non-Canonical Attributes of the UPR

As the intricacies of UPR biology are uncovered, it has become increasingly apparent that the role of UPR transducers extends beyond mere oversight of protein folding in the ER. Several studies demonstrate that the UPR is also potently induced in response to altered ER membrane lipid composition, referred to as ‘lipid bilayer stress’ [44]. Cell membrane receptor signaling, such as mTOR, protein kinase A (PKA) and toll-like receptor (TLR) signaling have been shown to engage IRE1 activity even in the absence of protein folding stress [21]. Recent studies have uncovered the role of IRE1 and PERK as a hub for inter-organellar crosstalk that regulates cytoskeleton remodeling and mitochondrial bioenergetics [21, 43]. Profiling of XBP1s targets in multiple tissues has uncovered a novel requirement of this transcription factor in modulating cell differentiation, hypoxia response, neuronal plasticity, angiogenesis, lipid metabolism, and glucose metabolism that is independent of ER stress [24, 45]. Indeed, the ability of the UPR to integrate varied and often dichotomous (i.e. adaptive and apoptotic) outputs renders it an ideal signaling framework to buffer physiologic fluctuations and facilitate cellular homeostasis [46].

### UPR In Skeletal Health

Many studies have reported a critical role of ER stress in cartilage biology [47]. The UPR has been implicated as a core mechanism in genetic skeletal diseases (GSDs). GSDs are a diverse and complex group of growth plate disorders resulting from mutations in genes encoding cartilaginous proteins such as cartilage oligomeric matrix protein, matrilin-3, and collagen types II and X. These studies have been summarized by Briggs and colleagues [48] and Rellmann and Dreier [49]. ER stress has also been implicated as a driver of articular cartilage degeneration in osteoarthritis in recent studies reviewed by Hughes and colleagues [50].

The most compelling proof that the UPR is important in osteoblast biology comes from the finding that patients with Wolcott-Rallison syndrome, who lack a functional PERK protein, exhibit skeletal dysplasia in addition to early onset of diabetes, growth retardation, cognitive defects, and early mortality [51]. One study described association of a haplotype of the *Eif2ak3* gene (encoding PERK) with low bone mineral density in separate cohorts of Amish and Mexican American subjects [52]. Patients with hypomorphic mutations in S1P protease or missense mutations in S2P protease, which disrupt its ability to cleave and activate ATF6 (in addition to other substrates), exhibit either skeletal dysplasia or a recessive form of osteogenesis imperfecta, respectively [53]. However, proteolytic activation of additional substrates such as sterol regulatory element-binding protein (SREBP) by these proteases precludes attributing the poor skeletal outcomes solely to the ATF6 substrate. Our query of UPR transducers in the Musculoskeletal Knowledge Portal (MSK-KP) [54] showed that variants in XBP1 were associated with heel bone mineral density (also known as estimated bone mineral density). These and other studies, reviewed by Horiuchi and colleagues [47], suggest that osteoblasts and osteoclasts rely on the UPR to actively regulate cell differentiation in addition to normalizing ER homeostasis. The following sections highlight salient features of the UPR in osteoblast and osteoclast biology and its implications to skeletal physiology.

#### Regulation of Osteoblast Biology by the UPR

All three axes of the UPR are robustly activated in response to osteogenic induction by BMP-2 in cell culture studies, and parallel the expression of *Runx2*, *Osx1/Sp7*, collagen (*Col1a1*) and osteocalcin (*Ocn*) genes [55–57]. A daily pulse-treatment with tunicamycin or thapsigargin, chemicals that induce ER stress by inhibiting N-glycosylation of proteins and raising cytosolic Ca^+2^ levels [58], stimulates the UPR and augmented the osteogenic expression profile [56, 59, 60]. Continued exposure to these UPR inducers, however, reduces cell viability and elicits apoptotic programs [59, 61]. Iyer et al. [62] reported that pharmacologic induction of UPR in cultures of primary osteoblastic cells, as well as in osteoblast (UAMS-32) and osteocyte (MLO-Y4) cell lines, increases RANKL expression. Indeed, administration of tunicamycin to mice elevated the UPR and RANKL expression in cortical bone and augmented bone resorption. Collectively, these studies demonstrate that mild ER stress aids osteogenic differentiation and extracellular matrix synthesis, but protracted ER stress hastens cell death.

The pro-osteogenic role of the UPR in osteoblast biology is further reinforced by evidence from genetic murine models. Akin to clinical subjects, mice with germline deletion of *Eif2ak3* (*Perk*^*−/−*^) exhibit low bone mass, albeit in backgrounds of system-wide tissue dysfunction and general poor health [63]. Furthermore, osteoblasts in *Perk*^*−/−*^ mice have defective collagen-processing capacity and a distended ER (a morphologic indicator of ER stress [58]), and exhibit reduced mineralization in cell cultures. Germline deletion of the *Atf4* gene also reduces murine bone mass. Mechanistically, ribosomal S6 kinase 2 (RSK2) is also a critical activator of ATF4 in osteoblasts and favors synthesis of collagen type I secondary to cellular import of amino acid [64]. Therefore, additional studies are warranted to conclusively establish a pro-osteogenic role for PERK in vivo.

Germline deletion of *Ire1α* (encoding IRE1) causes embryonic lethality [65]. One in vitro study reported that overexpression of IRE1 repressed osteoblastogenesis [66]. In contrast, studies in MC3T3-E1 cells demonstrate that IRE1/XBP1s and ATF6 promote transcription of *Osx1* and *Ocn,* respectively [56, 57]. Curiously, deletion of XBP1s in Col2-Cre expressing cells (Col2-Cre  marks osteogenic and chondrogenic cells [67]) led to a mild delay in mineralization of endochondral bones during development. No additional embryonic or postnatal skeletal defects were reported, suggesting that additional regulatory mechanisms likely offset requirement for the IRE/XBP1s/Osx1 axis in vivo*.* *ATF6*^−/−^ mice are viable; however, the contribution of this ER stress sensor in osteoblast biology has not yet been investigated directly [47]. Nonetheless, mice with ablation of S1P protease in osteoblasts exhibited vertebral and hindlimb defects [68]. It is also noteworthy that OASIS (Old Astrocyte Specifically Induced Substance), a bZIP ER resident transcription factor that shares structural similarity with ATF6, is highly expressed in osteoblasts [47]. Curiously, abrogation of OASIS inhibited bone formation and fracture healing in mice [69]. This study has implications for a favorable role of the UPR in skeletal repair. Nonetheless, a lack of comprehensive genetic studies precludes concluding that IRE1 and ATF6 signaling contribute to osteogenic differentiation in vivo.

#### Regulation of Bone Resorption by the UPR

A critical role of the UPR in regulating osteoclast activity is also emerging from several studies. IRE1/XBP1s and PERK/ATF4 axes have been reported to upregulate NFkB transcription during RANKL-induced osteoclast differentiation of bone marrow derived macrophages [70, 71]. Indeed, bone mass is augmented or reduced, respectively, in mice with conditional deletion of *Ire1α* or overexpression of *Atf4* in osteoclasts [70]. These findings raise the possibility of cooperative signaling between IRE1 and PERK axes during osteoclast differentiation. Additionally, PERK signaling can also support osteoclast activity by upregulating MAPK signaling, autophagy, F-actin ring formation, and expression of the osteoclast markers *TRAP*, *MMP9,* and *Cathepsin K* [72]. Overexpression of *ATF6* in bone marrow macrophages has been shown to induce NFATc1 and CHOP transcripts and to augment genesis and activity of osteoclasts, in part by stimulating autophagy [73] (discussed in detail in the next section). These findings suggest that UPR signaling can augment osteoclast differentiation and activity. Nonetheless, the in vivo roles of PERK and ATF6 in osteoclast biology have not yet been addressed directly.

Protracted phosphorylation of eIF2α, chemically induced by salubrinal [74], suppresses RANKL-induced osteoclast differentiation and activity [75, 76]. Salubrinal is an inhibitor of PP1, downstream of PERK and ISR kinases [74]. Salubrinal repressed NFATc1 expression in preosteoclasts, which was attributed at least in part to an overall reduction in translation efficiencies [75]. Additionally, salubrinal also inhibited migration and adhesion of osteoclasts by downregulating Rac1 GTPase activity [76].This finding may be attributed to prolonged inhibition of protein synthesis and an altered osteoclast proteome that likely impedes several aspects of osteoclastogenesis. Additional findings from these studies are discussed in the next section in the context of relevance to alleviating osteoporotic bone loss.

Taken together, these studies demonstrate that the UPR can modulate the commitment into osteoblast and osteoclast lineages in response to physiologic cues and pathologic insults. Engagement of homeostatic processes within the ER by the UPR fine-tune and expand their secretome during differentiation, thereby aiding their optimal function. In contrast, a maladaptive UPR has been implicated in the occurrence of skeletal dysplasia (reviewed in [48, 49]), osteosarcoma [77], osteoarthritis (reviewed in [50]), rheumatoid arthritis (reviewed in [78, 79]) and systemic lupus erythematosus (reviewed in [79]), and osteoporotic bone loss.

### UPR in the Pathogenesis of Osteoporosis

Osteoporosis is a skeletal disorder characterized by deficits in bone mass, compromised skeletal microarchitecture, and/or altered matrix composition that increases the risk of fracture [80–82]. Disruption of skeletal remodeling due to deficits in bone formation relative to resorption eventually lead to a net negative bone balance. Bone loss starts as early as the third decade of an individual’s life, irrespective of their sex and race [81, 83]. The US prevalence of osteoporosis and osteopenia in individuals > 50 years of age in 2010 was 10.3% and 43.9% with a predicted upward trend [83, 84]. In addition to advancing age, other prevalent factors such as estrogen withdrawal at menopause in women [81], therapeutic use of glucocorticoids [85, 86], and incidence of diabetes [87] are known to accelerate the development of osteoporosis. Findings that implicate the emerging contribution of the UPR to skeletal involution in these settings are summarized in this section.

#### Aging

Age-related osteoporosis, an inexorable companion of longevity (as described in [81]), poses an enormous health-care burden. One in 3 men and 1 in 5 women aged 50 years and older are projected to suffer an osteoporotic fracture in their lifetime [82]. A profound decline in bone formation and increased cortical porosity are key drivers of skeletal deterioration in the elderly [81, 82]. This trend will likely increase with increased life expectancy. Accumulation of senescent SSCs and osteocytes, osteoblast/osteocyte apoptosis, and increased resorption have been identified as core processes that underlie bone fragility.

Hino et al. [88] first noted that the osteoblasts from osteoporotic patients had a reduction in ER molecular chaperones, BiP and PDI (protein-disulfide isomerase) expression, and KDEL immunostaining in bone biopsies. The KDEL peptide sequence is critical for retention of proteins to the ER, including ER-resident chaperones, [89] and its reduction suggests deficits in secretory protein synthesis and/or protein folding capacity of aged osteoblasts. Osteocyte cultures obtained from aged (24–26 months) C57BL/6 J mice exhibited an increase in ATF4, XBP1s and CHOP transcripts [90]. The response to fluid flow stimuli, as determined by Cox2 expression and nitric oxide production, was also greatly diminished, suggesting that elevated UPR in osteocytes may underlie the deficits in responding to mechanical stimuli that occurs with aging.

Fragility of the aged bone is attributed, in part, to increases in advanced glycation end products (AGEs) that can alter collagen crosslinks [91]. AGEs are generated by non-enzymatic reaction between a reactive carbonyl group of a reducing sugar and amines on lipids or proteins. Many studies using human and mouse cell-lines have shown that exposure to AGEs inhibits osteoblastic differentiation and promotes apoptosis [60, 92–95]. Tanaka et al. [60] first reported that bone marrow-derived ST2 cells, induced to differentiate with BMP-2 and exposed to AGEs for 7 days (glycated bovine serum albumin), exhibited a reduction in IRE1 and ATF6 activity concomitant with suppression of osteogenic transcripts. This suggests that AGEs impair pro-osteogenic actions of the UPR transducers. In another study, one hour exposure to glycolaldehyde (an AGE intermediate) induced dissociation of IRE1 from BiP in calvaria-derived MC3T3-E1 osteoblasts and stimulated IRE1-pJNK/p38 signaling [95]. The divergent response of IRE1 signaling in these two studies [60, 95] might be attributed to temporal engagement of distinct IRE1 outputs. Protracted ER stress in the first study [60] likely repressed osteogenic outputs whereas an acute insult in the second study [95] stimulated the pro-apoptotic axis. Different concentrations and types of the AGE reagents used in the two studies also may have contributed to distinct responses. Despite the differences, both studies noted an increase in PERK signaling [60, 95]. Curiously, however, neither silencing *Perk* or *CHOP* mitigated the adverse effects of AGEs on differentiation or cell viability, respectively. Additional studies are needed to determine if IRE, and not PERK signaling, is causal in AGEs-associated osteoblast dysfunction with aging in vivo. Nonetheless, these studies support the notion that AGEs in the aged bone matrix may elicit ER stress in osteoblastic cells.

Illness or recovery from injury often involves long periods of immobility in the aged and can lead to bone loss. Hind-limb immobilization in mice (via tail suspension) induced ER dilation in osteoblasts, concurrent with apoptosis [75]. Furthermore, osteoblast cultures obtained from the bone marrow of these tail-suspended mice had reduced p-eIF2α, but elevated CHOP and RANKL protein levels. Administration of salubrinal, however, normalized unloading-induced reduction in osteoblast number and viability. Salubrinal also attenuated disuse-related resorption by inhibiting NFATc1 mediated osteoclastogenesis [75]. These findings suggest that bone loss in limb immobilization may in part be attributed to ER stress.

The evidence of UPR dysregulation in the context of skeletal aging as such is currently scarce. However, multiple studies described in subsequent sections show that ER stress is induced in the skeleton in the context of other settings that coexist in the elderly, including menopausal estrogen withdrawal, glucocorticoid excess, and diabetes.

#### Glucocorticoid-Related Osteoporosis

Long-term glucocorticoid (GC) therapy is prescribed for several conditions, including rheumatoid arthritis, asthma, organ transplantation, and as a component of cancer chemotherapy. Use of GCs is associated with a 30–50% increase in fracture risk in adult and pediatric populations [86]. Collapse of the femoral head due to disrupted blood supply is a debilitating sequalae that develops in 5–40% of patients prescribed GCs [85]. This condition is known as ischemic necrosis, avascular necrosis, or osteonecrosis. Osteonecrosis accounts for 10% of total hip replacement surgeries in the US and 2.8%–10% across Canadian, Swedish, and Australian registries [96, 97].

Bone biopsies from patients and preclinical models of GC excess present low bone formation and elevated resorption as well as femoral head edema [98–100]. Unfavorable skeletal outcomes associated with GCs are attributed partly to osteoblast and osteocyte apoptosis [98, 99]. Treatment with dexamethasone, an in vitro paradigm of GC excess [101], activates all 3 UPR transducers, and increases p-eIF2α, BiP, and CHOP levels concomitant with induction of proapoptotic signaling in MC3T3-E1 cells [102, 103]. Treatment with either salubrinal [61] or 4-phenyl butyric acid (4-PBA, a chemical chaperone that aids protein folding and alleviates ER stress [103, 104]), or silencing of CHOP [102] rescued the GC-induced apoptosis in osteoblastic cells. Together these studies support the concept that elevated UPR and subsequent CHOP-induced apoptosis of osteoblasts and/or osteocytes may contribute to GC-related bone fragility.

Biochemical indices of the UPR in the context of GC excess have not been investigated in vivo. Nonetheless, salubrinal attenuated the low bone mass and bone formation phenotypes associated with exogenous GC in mice [61], implicating ER stress as a possible mediator of adverse skeletal outcomes. The bone sparing effects of salubrinal were attributed to alleviation of osteoblast and osteocyte apoptosis in this study. However, inhibitory effects of salubrinal on bone resorption [75, 76] in GC excess cannot be ruled out. Akin to this study, Liu et al. [105] reported beneficial effects of salubrinal in a surgical model of ischemic osteonecrosis in mice. Increased resorption, together with low bone formation, leads to rapid loss of subchondral bone in early phases of osteonecrosis [100]. Salubrinal normalized the excess bone resorption by attenuating NFATc1 signaling in osteoclasts and increased osteoblast numbers in the osteonecrotic femoral head [105]. Additional in vivo evidence implicating PERK as a mediator of the adverse effects of GCs on the skeleton comes from another study wherein rats were treated with methylprednisolone to induce osteonecrosis [106]. Administration of the Perk inhibitor GSK2656157 protected development of osteonecrosis in rats despite treatment with methylprednisolone. However, the beneficial effects of GSK2656157 may, in part, be due to inhibition of receptor-interacting protein kinase 1 (RIPK1) [107], which has been implicated in the pathology of osteonecrosis [108]. Since the histomorphometric indices of bone turnover were not reported in this study [106], the in vivo cellular targets of GSK2656157 remain unclear. Notably, salubrinal and GSK2656157 also preserved vascularity of the subchondral bone in addition to the bone sparing effects [105, 106]. This latter observation suggests that elevated PERK activity in more than one cell type, including the endothelial cells, may contribute to the pathogenesis of osteonecrosis. Thus, additional studies are warranted to clarify the contribution of PERK in mediating adverse effects of GCs. Of note, the role of IRE1 and ATF6 signaling in GC-induced osteoporosis and osteonecrosis has not yet been explored in vivo or in vitro.

#### Post-Menopausal Osteoporosis

Most women experience a window of accelerated loss of bone mass and skeletal strength during menopause, when estrogen levels decline substantially following cessation of gonadal function. In multiple population studies, the incidence of fragility fractures in women is at least twice that of men, especially 50 years of age onwards [109–111]. Acute loss of estrogen increases bone resorption as well as formation, but the former outpaces the latter resulting in net loss of bone [109, 112].

Estrogens restrain resorption by inhibiting differentiation and promoting premature apoptosis of osteoclast progenitors [112, 113]. Inhibition of eIF2α activity, using salubrinal, prevented bone resorption and loss of cancellous bone in estrogen-deficient mice [76]. An altered osteoclast proteome due to prolonged translational arrest by salubrinal (see the section *Regulation of bone resorption by the UPR*), may underlie the deficits in differentiation and migration of osteoclasts in this study. Another study reported increased expression of the Golgi protease S1P in the osteoclasts of ovariectomized mice by immunostaining the femur sections [73]. Furthermore, deletion of S1P in LysM-Cre expressing macrophages abrogated ATF6 activity, as well as the ovariectomy-induced increase in osteoclast number, and preserved cancellous bone mass. Pharmacologic inhibition of S1P by PF429242 also prevented ovariectomy-induced bone loss. Mechanistically, S1P contributes to ATF6 and SREBP 2 (SREBP2) maturation by proteolytic cleavage. SREBP2 is  a transcription factor that can promote autophagy [114]. The authors [73] demonstrated that CHOP, induced by ATF6 in conjunction with SREBP2, stimulated autophagy in osteoclasts. Induction of autophagy is critical for bone resorption by osteoclasts [115]. Specifically, synergistic binding of CHOP and SREBP2 at the LC3 promoter stimulates its transcription [73]. LC3 is an essential component of the autophagosome and mediates actin ring formation, bone resorption, and release of cathepsin K by osteoclasts [116, 117]. These findings suggest that antiresorptive effects of estrogens on bone may result, at least in part, from suppression of eIF2α and ATF6 signaling in osteoclasts. Nonetheless, additional studies are warranted to delineate the molecular sequalae underlying estrogen-related induction of the UPR in osteoclasts.

Increased osteoblast and osteocyte apoptosis associated with estrogen deficiency may also contribute to osteoporosis [109, 112]. Substantial evidence suggests that estrogen maintains osteoblast viability, in part, by modulating ER stress responses. Guo et al. [118] reported that estradiol protected MC3T3-E1 osteoblasts against thapsigargin-induced apoptosis by increasing recruitment of transcription factor TFII-I to the BiP promoter and stimulating its transcription [118]. The authors implicate BiP in conferring protection to ER stress induced cell death. In addition to aiding protein folding, BiP can confer protection from apoptosis by complexing with procaspases or binding proapoptotic BCL-2-interacting killer (BIK) [119]. The latter interaction sequesters BIK to the ER and relieves the inhibition on BCL-2, leading to suppression of Ca^+2^ release from the ER. Immunostaining bone biopsies obtained from post-menopausal osteoporotic subjects revealed that expression of ER molecular chaperones, BiP and PDI (Protein-disulfide isomerase), was down regulated in osteoblasts [88]. Furthermore, administration of BiX, a selective activator of BiP, protected ovariectomy-induced loss of BMD in mice [88]. Li and colleagues [76] reported that osteoblasts from ovariectomized mice exhibited dilated ER in electron micrographs, concurrent with reduction in autophagosomes. In addition to elevated p-eIF2α and CHOP levels, cultures of bone marrow-derived osteoblasts from estrogen-deficient mice had reduced p62 and LC3I to LCII conversion [76]. These findings substantiate the notion that autophagy was also compromised in addition to UPR dysregulation. Salubrinal normalized the aberrant UPR and improved autophagy in osteoblasts of estrogen-deficient mice. Collectively, these studies suggest that correcting ER proteostasis may prolong osteoblast lifespan in the setting of estrogen withdrawal, and alleviate postmenopausal bone loss.

Estrogens restrain expression of pro-osteoclastogenic factors by cells of the osteoblast lineage, but the evidence of their being direct targets of estrogen is lacking [120]. Studies described above have not investigated if excessive UPR contributes to the altered osteoblast and/or osteocyte secretome that is associated with estrogen depletion. In support of this concept, elevated UPR can stimulate production of RANKL by osteoblasts and osteocytes [62]. Future work aiming to elucidate the underlying molecular mechanisms may yield critical insights toward optimizing therapeutics for osteoporosis.

#### Diabetes

Diabetes mellitus (DM) is characterized by poor glycemic control due to either inadequate insulin production by the pancreatic β-cells (type 1 diabetes; T1DM) or failure to compensate for insulin resistance (type 2 diabetes; T2DM). Despite clear differences in disease etiology, ER stress and UPR dysregulation have emerged as one of the shared and pivotal contributors to the pathogenesis of T1DM and T2DM [121–124]. A collapse of ER proteostasis is a significant contributor of insulin misfolding and β-cell dysfunction in T1DM [121, 125]. In the context of T2DM, overwhelming evidence suggests that a combination of inflammation and ER stress, triggered in response to systemic increase in glucose and free fatty acid, are key drivers of insulin resistance [123, 124].

Given the increased prevalence of diabetes, poor bone health and a higher risk of fracture in diabetics have become significant clinical concerns in recent years [126, 127]. Delayed union and non-union are more frequent in diabetic patients who experience a fracture. Patients with T1DM or T2DM have low levels of osteocalcin, a marker of bone formation, suggesting that osteogenic deficits contribute in part to the skeletal fragility [128]. Multiple in vitro studies have demonstrated the adverse effects of hyperglycemia on osteoblast maturation and expression of osteocalcin [129–131]. Liu et al. [132] reported that high glucose stimulated expression of the proapoptotic CHOP in cultured calvarial osteoblasts. In the same study, femoral sections from streptozotocin-injected rats, which mimic T1D, exhibited increased CHOP immunostaining in osteoblasts compared to non-diabetic controls. Mice overexpressing CHOP under the control of the human osteocalcin promoter had reduced bone formation secondary to increased osteoblast apoptosis [133]. Collectively, these studies suggest that low bone mass in T1DM may, in part, be attributed to CHOP-mediated osteoblast apoptosis.

Obesity increases the predisposition to develop T2D in part by eliciting ER stress [21, 122]. Additionally, the detrimental effects of obesity on the skeleton and associated fracture risk are increasingly appreciated [134–137]. Adipocytic skewing of the bone marrow resident SSCs is thought to contribute to skeletal fragility. In support of this notion, SSCs isolated from the bone marrow of obese mice and individuals with BMI > 35 both exhibited a preferential shift toward an adipocytic transcriptome concomitant with senescence, as determined by increased intracellular ROS and senescence-associated marker β-galactosidase activity [138–140]. An independent study by Ulum et al. [141] demonstrated that SSCs obtained from individuals with high BMI had elevated expression of ATF4 and CHOP transcripts, concurrent with the suppressed osteogenic response. Although XBP1 mRNA showed a trend to decrease in this study, it was not statistically significant. Moreover, the treatment with ER stress attenuating chemical chaperones, TUDCA and 4-PBA [104], alleviated the osteogenic deficits of SSCs obtained from high-BMI individuals by partially normalizing the UPR. Thus, an aberrant UPR in SSCs may drive some of the osteogenic deficits in diabetes-related skeletal fragility and fracture repair.

Hyperglycemia also fosters increased production of AGEs in the collagenous bone matrix of DM patients [142]. AGE accumulation makes the bone brittle and contributes to its fragility. The occurrence of AGEs within fracture callus noted by Khajuria et al. [143] may contribute to delayed ossification and healing in obese mice. In agreement with this possibility, high glucose exacerbated the adverse effects of AGEs on osteocalcin expression and osteoblast differentiation of MC3T3-E1 cells [144]. However, the combinatorial effects of glucose and AGEs on the UPR within the osteogenic lineage have not been investigated to date.

## Conclusions and Perspective

The ability of the UPR to affect diametrically opposite cellular outcomes- survival versus cell death- solidifies its role as a critical determinant of cell fate in many tissues. The studies described in this review favor extending this concept to the skeleton. Nonetheless, a comprehensive understanding of UPR biology of the skeleton is lacking. Several studies, referenced herein, have deduced the molecular underpinnings of ER stress sensors in osteoblast and osteoclast differentiation using cell culture systems. As a natural progression, verifying their relevance to skeletal physiology in vivo is essential. Parsing the contribution of distinct UPR sensors and their crosstalk in skeletal physiology in vivo is a critical first step to addressing UPR dysregulation in skeletal pathologies. The PERK axis has been investigated extensively in osteoporotic paradigms, albeit via p-eIF2α. However, the role of ER stress in causation of bone loss is equivocal, as eIF2α also integrates other stress responses that may be affected with osteoporosis. The possibility of crosstalk (either antagonistic or complementary) between various UPR transducers in mediating skeletal health is another aspect that requires further research.

Over the past decade, the UPR has gained prominence as a druggable target for several pathologies, including neurodegenerative diseases, metabolic disorders, and cancer [74, 145]. Salubrinal and chemical chaperones are reported to have alleviated adverse skeletal outcomes in rodent models [61, 75, 76, 105, 106, 146–150]. There has been a surge in identification of small molecule compounds that target specific aspects of the UPR interactome [74, 145]. These molecules offer improved pharmacokinetics, are well tolerated by mice, and in preclinical studies show promise in curbing the terminal signaling associated with several diseases. Optimizing these therapeutics as treatment modalities for skeletal diseases provides the impetus to gain a greater understanding of UPR biology as it pertains to skeletal physiology and pathology.

## References

[1] Arora D, Robey PG (2022). Recent updates on the biological basis of heterogeneity in bone marrow stromal cells/skeletal stem cells. Biomater Transl.

[2] Robey P (2017) "Mesenchymal stem cells": fact or fiction, and implications in their therapeutic use. F1000Res. 6, 524 10.12688/f1000research.10955.1 PMC5399967 28491279

[3] Robey PG, Riminucci M, Bilezikian JP, Martin TJ, Clemens TL, Rosen CJ (2020). Chapter 2 - Skeletal stem cells: Tissue-specific stem/progenitor cells of cartilage, bone, stroma, and marrow adipocytes. Principles of Bone Biology (Fourth Edition).

[4] de Gorter DJJ, Sánchez-Duffhues G, ten Dijke P (2018) Signal Transduction Cascades Controlling Osteoblast Differentiation. In: John P. Bilezikian, Roger Bouillon, Thomas Clemens, Juliet Compston, Douglas C. Bauer, Peter R. Ebeling, Klaus Engelke, David Goltzman, Theresa Guise, Suzanne M. Beur, Harald Jüppner, Karen Lyons, Laurie McCauley, Michael R. McClung, Paul D. Miller, Socrates E. Papapoulos, G. David Roodman, Clifford J. Rosen, Ego Seeman, Rajesh V. Thakker, Michael P. Whyte, Mone Zaidi (Eds.) Primer on the Metabolic Bone Diseases and Disorders of Mineral Metabolism. Wiley, NJ. p 54–59

[5] Godman GC, Porter KR (1960). Chondrogenesis, studied with the electron microscope. J Biophys Biochem Cytol.

[6] Roy S, Meachim G (1968). Chondrocyte ultrastructure in adult human articular cartilage. Ann Rheum Dis.

[7] Barnett CH, Cochrane W, Palfrey AJ (1963). Age changes in articular cartilage of rabbits. Ann Rheum Dis.

[8] Silberberg M, Silberberg R, Hasler M (1964). Ultrastructure of articular cartilage of mice treated with somatotrophin. J Bone Joint Surg Am.

[9] Davies DV, Barnett CH, Cochrane W, Palfrey AJ (1962). Electron microscopy of articular cartilage in the young adult rabbit. Ann Rheum Dis.

[10] Robling AG, Bonewald LF (2020). The osteocyte: new insights. Annu Rev Physiol.

[11] Kitase Y, Prideaux M (2023). Regulation of the osteocyte secretome with aging and disease. Calcif Tissue Int.

[12] Boyle WJ, Simonet WS, Lacey DL (2003). Osteoclast differentiation and activation. Nature.

[13] Takayanagi H (2021). RANKL as the master regulator of osteoclast differentiation. J Bone Miner Metab.

[14] Braakman I, Bulleid NJ (2011). Protein folding and modification in the mammalian endoplasmic reticulum. Annu Rev Biochem.

[15] Lam AK, Galione A (2013). The endoplasmic reticulum and junctional membrane communication during calcium signaling. Biochim Biophys Acta.

[16] Wang L, Wang CC (2022). Oxidative protein folding fidelity and redoxtasis in the endoplasmic reticulum. Trends Biochem Sci.

[17] Hwang J, Qi L (2018). Quality control in the endoplasmic reticulum: crosstalk between ERAD and UPR pathways. Trends Biochem Sci.

[18] Wiseman RL, Mesgarzadeh JS, Hendershot LM (2022). Reshaping endoplasmic reticulum quality control through the unfolded protein response. Mol Cell.

[19] Ferro-Novick S, Reggiori F, Brodsky JL (2021). ER-Phagy, ER homeostasis, and ER quality control: implications for disease. Trends Biochem Sci.

[20] Dikic I, Elazar Z (2018). Mechanism and medical implications of mammalian autophagy. Nat Rev Mol Cell Biol.

[21] Hetz C, Zhang K, Kaufman RJ (2020). Mechanisms, regulation and functions of the unfolded protein response. Nat Rev Mol Cell Biol.

[22] Wu J, Kaufman RJ (2006). From acute ER stress to physiological roles of the Unfolded Protein Response. Cell Death Differ.

[23] Walter P, Ron D (2011). The unfolded protein response: from stress pathway to homeostatic regulation. Science.

[24] Hetz C, Papa FR (2018). The unfolded protein response and cell fate control. Mol Cell.

[25] Chen Y, Brandizzi F (2013). IRE1: ER stress sensor and cell fate executor. Trends Cell Biol.

[26] Glembotski CC, Rosarda JD, Wiseman RL (2019). Proteostasis and beyond: ATF6 in ischemic disease. Trends Mol Med.

[27] Karagoz GE, Acosta-Alvear D, Walter P (2019). The unfolded protein response: detecting and responding to fluctuations in the protein-folding capacity of the endoplasmic reticulum. Cold Spring Harb Perspect Biol.

[28] Hetz C, Chevet E, Oakes SA (2015). Proteostasis control by the unfolded protein response. Nat Cell Biol.

[29] Preissler S, Ron D (2019). Early events in the endoplasmic reticulum unfolded protein response. Cold Spring Harb Perspect Biol.

[30] Wek RC (2018) Role of eIF2alpha Kinases in Translational Control and Adaptation to Cellular Stress. Cold Spring Harb Perspect Biol 10 10.1101/cshperspect.a032870 PMC6028073 29440070

[31] Vattem KM, Wek RC (2004). Reinitiation involving upstream ORFs regulates ATF4 mRNA translation in mammalian cells. Proc Natl Acad Sci U S A.

[32] Harding HP, Zhang Y, Zeng H, Novoa I, Lu PD, Calfon M, Sadri N, Yun C, Popko B, Paules R, Stojdl DF, Bell JC, Hettmann T, Leiden JM, Ron D (2003). An integrated stress response regulates amino acid metabolism and resistance to oxidative stress. Mol Cell.

[33] Novoa I, Zhang Y, Zeng H, Jungreis R, Harding HP, Ron D (2003). Stress-induced gene expression requires programmed recovery from translational repression. EMBO J.

[34] Lee AH, Iwakoshi NN, Glimcher LH (2003). XBP-1 regulates a subset of endoplasmic reticulum resident chaperone genes in the unfolded protein response. Mol Cell Biol.

[35] Maurel M, Chevet E, Tavernier J, Gerlo S (2014). Getting RIDD of RNA: IRE1 in cell fate regulation. Trends Biochem Sci.

[36] Sandow JJ, Dorstyn L, O'Reilly LA, Tailler M, Kumar S, Strasser A, Ekert PG (2014). ER stress does not cause upregulation and activation of caspase-2 to initiate apoptosis. Cell Death Differ.

[37] Hetz C (2012). The unfolded protein response: controlling cell fate decisions under ER stress and beyond. Nat Rev Mol Cell Biol.

[38] Teske BF, Wek SA, Bunpo P, Cundiff JK, McClintick JN, Anthony TG, Wek RC (2011). The eIF2 kinase PERK and the integrated stress response facilitate activation of ATF6 during endoplasmic reticulum stress. Mol Biol Cell.

[39] Yoshida H, Uemura A, Mori K (2009). pXBP1(U), a negative regulator of the unfolded protein response activator pXBP1(S), targets ATF6 but not ATF4 in proteasome-mediated degradation. Cell Struct Funct.

[40] Yoshida H, Oku M, Suzuki M, Mori K (2006). pXBP1(U) encoded in XBP1 pre-mRNA negatively regulates unfolded protein response activator pXBP1(S) in mammalian ER stress response. J Cell Biol.

[41] Gonen N, Sabath N, Burge CB, Shalgi R (2019). Widespread PERK-dependent repression of ER targets in response to ER stress. Sci Rep.

[42] Chang TK, Lawrence DA, Lu M, Tan J, Harnoss JM, Marsters SA, Liu P, Sandoval W, Martin SE, Ashkenazi A (2018). Coordination between two branches of the unfolded protein response determines apoptotic cell fate. Mol Cell.

[43] Karagoz GE, Aragon T, Acosta-Alvear D (2019) Recent advances in signal integration mechanisms in the unfolded protein response. F1000Res 8, 1840 10.12688/f1000research.19848.1 PMC6833987 31723416

[44] Halbleib K, Pesek K, Covino R, Hofbauer HF, Wunnicke D, Hanelt I, Hummer G, Ernst R (2017). Activation of the unfolded protein response by lipid bilayer stress. Mol Cell.

[45] Park SM, Kang TI, So JS (2021). Roles of XBP1s in transcriptional regulation of target genes. Biomedicines.

[46] Rutkowski DT, Hegde RS (2010). Regulation of basal cellular physiology by the homeostatic unfolded protein response. J Cell Biol.

[47] Horiuchi K, Tohmonda T, Morioka H (2016). The unfolded protein response in skeletal development and homeostasis. Cell Mol Life Sci.

[48] Briggs MD, Dennis EP, Dietmar HF, Pirog KA (2020) New developments in chondrocyte ER stress and related diseases. F1000Res 9 10.12688/f1000research.22275.1 PMC7194456 32399188

[49] Rellmann Y, Dreier R (2018). Different forms of ER stress in chondrocytes result in short stature disorders and degenerative cartilage diseases: new insights by cartilage-specific ERp57 knockout mice. Oxid Med Cell Longev.

[50] Hughes A, Oxford AE, Tawara K, Jorcyk CL, Oxford JT (2017). Endoplasmic reticulum stress and unfolded protein response in cartilage pathophysiology contributing factors to apoptosis and osteoarthritis. Int J Mol Sci.

[51] Julier C, Nicolino M (2010). Wolcott-Rallison syndrome. Orphanet J Rare Dis.

[52] Liu J, Hoppman N, O'Connell JR, Wang H, Streeten EA, McLenithan JC, Mitchell BD, Shuldiner AR (2012). A functional haplotype in EIF2AK3, an ER stress sensor, is associated with lower bone mineral density. J Bone Miner Res.

[53] Lindert U, Cabral WA, Ausavarat S, Tongkobpetch S, Ludin K, Barnes AM, Yeetong P, Weis M, Krabichler B, Srichomthong C, Makareeva EN, Janecke AR, Leikin S, Rothlisberger B, Rohrbach M, Kennerknecht I, Eyre DR, Suphapeetiporn K, Giunta C, Marini JC, Shotelersuk V (2016). MBTPS2 mutations cause defective regulated intramembrane proteolysis in X-linked osteogenesis imperfecta. Nat Commun.

[54] Kiel DP, Kemp JP, Rivadeneira F, Westendorf JJ, Karasik D, Duncan EL, Imai Y, Muller R, Flannick J, Bonewald L, Burtt N (2020). The musculoskeletal knowledge portal: making omics data useful to the broader scientific community. J Bone Miner Res.

[55] Saito A, Ochiai K, Kondo S, Tsumagari K, Murakami T, Cavener DR, Imaizumi K (2011). Endoplasmic reticulum stress response mediated by the PERK-eIF2(alpha)-ATF4 pathway is involved in osteoblast differentiation induced by BMP2. J Biol Chem.

[56] Tohmonda T, Miyauchi Y, Ghosh R, Yoda M, Uchikawa S, Takito J, Morioka H, Nakamura M, Iwawaki T, Chiba K, Toyama Y, Urano F, Horiuchi K (2011). The IRE1alpha-XBP1 pathway is essential for osteoblast differentiation through promoting transcription of Osterix. EMBO Rep.

[57] Jang WG, Kim EJ, Kim DK, Ryoo HM, Lee KB, Kim SH, Choi HS, Koh JT (2012). BMP2 protein regulates osteocalcin expression via Runx2-mediated Atf6 gene transcription. J Biol Chem.

[58] Oslowski CM, Urano F (2011). Measuring ER stress and the unfolded protein response using mammalian tissue culture system. Methods Enzymol.

[59] Hamamura K, Yokota H (2007). Stress to endoplasmic reticulum of mouse osteoblasts induces apoptosis and transcriptional activation for bone remodeling. FEBS Lett.

[60] Tanaka K, Yamaguchi T, Kaji H, Kanazawa I, Sugimoto T (2013). Advanced glycation end products suppress osteoblastic differentiation of stromal cells by activating endoplasmic reticulum stress. Biochem Biophys Res Commun.

[61] Sato AY, Tu X, McAndrews KA, Plotkin LI, Bellido T (2015). Prevention of glucocorticoid induced-apoptosis of osteoblasts and osteocytes by protecting against endoplasmic reticulum (ER) stress in vitro and in vivo in female mice. Bone.

[62] Iyer S, Melendez-Suchi C, Han L, Baldini G, Almeida M, Jilka RL (2020). Elevation of the unfolded protein response increases RANKL expression. FASEB Bioadv.

[63] Wei J, Sheng X, Feng D, McGrath B, Cavener DR (2008). PERK is essential for neonatal skeletal development to regulate osteoblast proliferation and differentiation. J Cell Physiol.

[64] Yang X, Matsuda K, Bialek P, Jacquot S, Masuoka HC, Schinke T, Li L, Brancorsini S, Sassone-Corsi P, Townes TM, Hanauer A, Karsenty G (2004). ATF4 is a substrate of RSK2 and an essential regulator of osteoblast biology; implication for Coffin-Lowry Syndrome. Cell.

[65] Reimold AM, Etkin A, Clauss I, Perkins A, Friend DS, Zhang J, Horton HF, Scott A, Orkin SH, Byrne MC, Grusby MJ, Glimcher LH (2000). An essential role in liver development for transcription factor XBP-1. Genes Dev.

[66] Guo FJ, Jiang R, Xiong Z, Xia F, Li M, Chen L, Liu CJ (2014). IRE1a constitutes a negative feedback loop with BMP2 and acts as a novel mediator in modulating osteogenic differentiation. Cell Death Dis.

[67] Ono N, Ono W, Nagasawa T, Kronenberg HM (2014). A subset of chondrogenic cells provides early mesenchymal progenitors in growing bones. Nat Cell Biol.

[68] Achilleos A, Huffman NT, Marcinkiewicyz E, Seidah NG, Chen Q, Dallas SL, Trainor PA, Gorski JP (2015). MBTPS1/SKI-1/S1P proprotein convertase is required for ECM signaling and axial elongation during somitogenesis and vertebral developmentdagger. Hum Mol Genet.

[69] Funamoto T, Sekimoto T, Murakami T, Kurogi S, Imaizumi K, Chosa E (2011). Roles of the endoplasmic reticulum stress transducer OASIS in fracture healing. Bone.

[70] Tohmonda T, Yoda M, Iwawaki T, Matsumoto M, Nakamura M, Mikoshiba K, Toyama Y, Horiuchi K (2015). IRE1alpha/XBP1-mediated branch of the unfolded protein response regulates osteoclastogenesis. J Clin Invest.

[71] Cao H, Yu S, Yao Z, Galson DL, Jiang Y, Zhang X, Fan J, Lu B, Guan Y, Luo M, Lai Y, Zhu Y, Kurihara N, Patrene K, Roodman GD, Xiao G (2010). Activating transcription factor 4 regulates osteoclast differentiation in mice. J Clin Invest.

[72] Guo J, Ren R, Sun K, Yao X, Lin J, Wang G, Guo Z, Xu T, Guo F (2020). PERK controls bone homeostasis through the regulation of osteoclast differentiation and function. Cell Death Dis.

[73] Zheng Z, Zhang X, Huang B, Liu J, Wei X, Shan Z, Wu H, Feng Z, Chen Y, Fan S, Zhao F, Chen J (2021). Site-1 protease controls osteoclastogenesis by mediating LC3 transcription. Cell Death Differ.

[74] Gonzalez-Teuber V, Albert-Gasco H, Auyeung VC, Papa FR, Mallucci GR, Hetz C (2019). Small molecules to improve ER proteostasis in disease. Trends Pharmacol Sci.

[75] Li J, Yang S, Li X, Liu D, Wang Z, Guo J, Tan N, Gao Z, Zhao X, Zhang J, Gou F, Yokota H, Zhang P (2017). Role of endoplasmic reticulum stress in disuse osteoporosis. Bone.

[76] Li J, Li X, Liu D, Hamamura K, Wan Q, Na S, Yokota H, Zhang P (2019). eIF2alpha signaling regulates autophagy of osteoblasts and the development of osteoclasts in OVX mice. Cell Death Dis.

[77] Yarapureddy S, Abril J, Foote J, Kumar S, Asad O, Sharath V, Faraj J, Daniel D, Dickman P, White-Collins A, Hingorani P, Sertil AR (2019). ATF6alpha activation enhances survival against chemotherapy and serves as a prognostic indicator in osteosarcoma. Neoplasia.

[78] Rahmati M, Moosavi MA, McDermott MF (2018). ER stress: a therapeutic target in rheumatoid arthritis?. Trends Pharmacol Sci.

[79] Miglioranza Scavuzzi B, Holoshitz J (2022) Endoplasmic Reticulum Stress, Oxidative Stress, and Rheumatic Diseases. Antioxidants (Basel) 11 10.3390/antiox11071306 PMC9312221 35883795

[80] Khosla S (2013). Pathogenesis of age-related bone loss in humans. J Gerontol A Biol Sci Med Sci.

[81] Manolagas SC (2018). The quest for osteoporosis mechanisms and rational therapies: how far we've come, how much further we need to go. J Bone Miner Res.

[82] Sfeir JG, Drake MT, Khosla S, Farr JN (2022). Skeletal aging. Mayo Clin Proc.

[83] Looker AC, Wahner HW, Dunn WL, Calvo MS, Harris TB, Heyse SP, Johnston CC, Lindsay R (1998). Updated data on proximal femur bone mineral levels of US adults. Osteoporos Int.

[84] Wright NC, Looker AC, Saag KG, Curtis JR, Delzell ES, Randall S, Dawson-Hughes B (2014). The recent prevalence of osteoporosis and low bone mass in the United States based on bone mineral density at the femoral neck or lumbar spine. J Bone Miner Res.

[85] Weinstein RS (2012). Glucocorticoid-induced osteoporosis and osteonecrosis. Endocrinol Metab Clin North Am.

[86] Weinstein RS (2011). Clinical practice. Glucocorticoid-induced bone disease. N Engl J Med.

[87] Hofbauer LC, Busse B, Eastell R, Ferrari S, Frost M, Muller R, Burden AM, Rivadeneira F, Napoli N, Rauner M (2022). Bone fragility in diabetes: novel concepts and clinical implications. Lancet Diabetes Endocrinol.

[88] Hino S, Kondo S, Yoshinaga K, Saito A, Murakami T, Kanemoto S, Sekiya H, Chihara K, Aikawa Y, Hara H, Kudo T, Sekimoto T, Funamoto T, Chosa E, Imaizumi K (2010). Regulation of ER molecular chaperone prevents bone loss in a murine model for osteoporosis. J Bone Miner Metab.

[89] Stornaiuolo M, Lotti LV, Borgese N, Torrisi MR, Mottola G, Martire G, Bonatti S (2003). KDEL and KKXX retrieval signals appended to the same reporter protein determine different trafficking between endoplasmic reticulum, intermediate compartment, and Golgi complex. Mol Biol Cell.

[90] Chalil S, Jaspers RT, Manders RJ, Klein-Nulend J, Bakker AD, Deldicque L (2015). Increased endoplasmic reticulum stress in mouse osteocytes with aging alters Cox-2 response to mechanical stimuli. Calcif Tissue Int.

[91] Saito M, Marumo K (2015). Effects of collagen crosslinking on bone material properties in health and disease. Calcif Tissue Int.

[92] Franke S, Siggelkow H, Wolf G, Hein G (2007). Advanced glycation endproducts influence the mRNA expression of RAGE, RANKL and various osteoblastic genes in human osteoblasts. Arch Physiol Biochem.

[93] Alikhani M, Alikhani Z, Boyd C, MacLellan CM, Raptis M, Liu R, Pischon N, Trackman PC, Gerstenfeld L, Graves DT (2007). Advanced glycation end products stimulate osteoblast apoptosis via the MAP kinase and cytosolic apoptotic pathways. Bone.

[94] McCarthy AD, Etcheverry SB, Cortizo AM (1999). Advanced glycation endproduct-specific receptors in rat and mouse osteoblast-like cells: regulation with stages of differentiation. Acta Diabetol.

[95] Suzuki R, Fujiwara Y, Saito M, Arakawa S, Shirakawa JI, Yamanaka M, Komohara Y, Marumo K, Nagai R (2020). Intracellular accumulation of advanced glycation end products induces osteoblast apoptosis via endoplasmic reticulum stress. J Bone Miner Res.

[96] Petek D, Hannouche D, Suva D (2019). Osteonecrosis of the femoral head: pathophysiology and current concepts of treatment. EFORT Open Rev.

[97] Moya-Angeler J, Gianakos AL, Villa JC, Ni A, Lane JM (2015). Current concepts on osteonecrosis of the femoral head. World J Orthop.

[98] Weinstein RS, Jilka RL, Parfitt AM, Manolagas SC (1998). Inhibition of osteoblastogenesis and promotion of apoptosis of osteoblasts and osteocytes by glucocorticoids. Potential mechanisms of their deleterious effects on bone. J Clin Invest.

[99] Weinstein RS, Nicholas RW, Manolagas SC (2000). Apoptosis of osteocytes in glucocorticoid-induced osteonecrosis of the hip. J Clin Endocrinol Metab.

[100] Weinstein RS, Hogan EA, Borrelli MJ, Liachenko S, O'Brien CA, Manolagas SC (2017). The pathophysiological sequence of glucocorticoid-induced osteonecrosis of the femoral head in male mice. Endocrinology.

[101] Frenkel B, White W, Tuckermann J (2015). Glucocorticoid-induced osteoporosis. Adv Exp Med Biol.

[102] Guo Y, Hao D, Hu H (2021). High doses of dexamethasone induce endoplasmic reticulum stress-mediated apoptosis by promoting calcium ion influx-dependent CHOP expression in osteoblasts. Mol Biol Rep.

[103] Yang J, Wu Q, Lv J, Nie H (2017). 4-Phenyl butyric acid prevents glucocorticoid-induced osteoblast apoptosis by attenuating endoplasmic reticulum stress. J Bone Miner Metab.

[104] Engin F, Hotamisligil GS (2010). Restoring endoplasmic reticulum function by chemical chaperones: an emerging therapeutic approach for metabolic diseases. Diabetes Obes Metab.

[105] Liu D, Zhang Y, Li X, Li J, Yang S, Xing X, Fan G, Yokota H, Zhang P (2017). eIF2alpha signaling regulates ischemic osteonecrosis through endoplasmic reticulum stress. Sci Rep.

[106] Gao Y, Zhu H, Wang Q, Feng Y, Zhang C (2020). Inhibition of PERK signaling prevents against glucocorticoid-induced endotheliocyte apoptosis and osteonecrosis of the femoral head. Int J Biol Sci.

[107] Rojas-Rivera D, Delvaeye T, Roelandt R, Nerinckx W, Augustyns K, Vandenabeele P, Bertrand MJM (2017). When PERK inhibitors turn out to be new potent RIPK1 inhibitors: critical issues on the specificity and use of GSK2606414 and GSK2656157. Cell Death Differ.

[108] Fan X, Xu X, Wu X, Xia R, Gao F, Zhang Q, Sun W (2022). The protective effect of DNA aptamer on osteonecrosis of the femoral head by alleviating TNF-alpha-mediated necroptosis via RIP1/RIP3/MLKL pathway. J Orthop Translat.

[109] Eastell R, O'Neill TW, Hofbauer LC, Langdahl B, Reid IR, Gold DT, Cummings SR (2016). Postmenopausal osteoporosis. Nat Rev Dis Primers.

[110] van Staa TP, Dennison EM, Leufkens HG, Cooper C (2001). Epidemiology of fractures in England and Wales. Bone.

[111] Pasco JA, Lane SE, Brennan-Olsen SL, Holloway KL, Timney EN, Bucki-Smith G, Morse AG, Dobbins AG, Williams LJ, Hyde NK, Kotowicz MA (2015). The epidemiology of incident fracture from cradle to senescence. Calcif Tissue Int.

[112] Almeida M, Laurent MR, Dubois V, Claessens F, O'Brien CA, Bouillon R, Vanderschueren D, Manolagas SC (2017). Estrogens and androgens in skeletal physiology and pathophysiology. Physiol Rev.

[113] Kim HN, Ponte F, Nookaew I, Ucer Ozgurel S, Marques-Carvalho A, Iyer S, Warren A, Aykin-Burns N, Krager K, Sardao VA, Han L, de Cabo R, Zhao H, Jilka RL, Manolagas SC, Almeida M (2020). Estrogens decrease osteoclast number by attenuating mitochondria oxidative phosphorylation and ATP production in early osteoclast precursors. Sci Rep.

[114] Seo YK, Jeon TI, Chong HK, Biesinger J, Xie X, Osborne TF (2011). Genome-wide localization of SREBP-2 in hepatic chromatin predicts a role in autophagy. Cell Metab.

[115] Montaseri A, Giampietri C, Rossi M, Riccioli A, Del Fattore A, Filippini A (2020). The role of autophagy in osteoclast differentiation and bone resorption function. Biomolecules.

[116] Chung YH, Yoon SY, Choi B, Sohn DH, Yoon KH, Kim WJ, Kim DH, Chang EJ (2012). Microtubule-associated protein light chain 3 regulates Cdc42-dependent actin ring formation in osteoclast. Int J Biochem Cell Biol.

[117] DeSelm CJ, Miller BC, Zou W, Beatty WL, van Meel E, Takahata Y, Klumperman J, Tooze SA, Teitelbaum SL, Virgin HW (2011). Autophagy proteins regulate the secretory component of osteoclastic bone resorption. Dev Cell.

[118] Guo YS, Sun Z, Ma J, Cui W, Gao B, Zhang HY, Han YH, Hu HM, Wang L, Fan J, Yang L, Tang J, Luo ZJ (2014). 17beta-Estradiol inhibits ER stress-induced apoptosis through promotion of TFII-I-dependent Grp78 induction in osteoblasts. Lab Invest.

[119] Lee AS (2014). Glucose-regulated proteins in cancer: molecular mechanisms and therapeutic potential. Nat Rev Cancer.

[120] Weitzmann MN, Pacifici R (2006). Estrogen deficiency and bone loss: an inflammatory tale. J Clin Invest.

[121] Sahin GS, Lee H, Engin F (2021). An accomplice more than a mere victim: The impact of beta-cell ER stress on type 1 diabetes pathogenesis. Mol Metab.

[122] Back SH, Kaufman RJ (2012). Endoplasmic reticulum stress and type 2 diabetes. Annu Rev Biochem.

[123] Eizirik DL, Cardozo AK, Cnop M (2008). The role for endoplasmic reticulum stress in diabetes mellitus. Endocr Rev.

[124] Cnop M, Foufelle F, Velloso LA (2012). Endoplasmic reticulum stress, obesity and diabetes. Trends Mol Med.

[125] Engin F (2016). ER stress and development of type 1 diabetes. J Investig Med.

[126] Bonds DE, Larson JC, Schwartz AV, Strotmeyer ES, Robbins J, Rodriguez BL, Johnson KC, Margolis KL (2006). Risk of fracture in women with type 2 diabetes: the Women's Health Initiative Observational Study. J Clin Endocrinol Metab.

[127] Sellmeyer DE, Civitelli R, Hofbauer LC, Khosla S, Lecka-Czernik B, Schwartz AV (2016). Skeletal metabolism, fracture risk, and fracture outcomes in type 1 and type 2 diabetes. Diabetes.

[128] Napoli N, Chandran M, Pierroz DD, Abrahamsen B, Schwartz AV, Ferrari SL, Bone IOF, Diabetes Working G (2017). Mechanisms of diabetes mellitus-induced bone fragility. Nat Rev Endocrinol.

[129] Zayzafoon M, Stell C, Irwin R, McCabe LR (2000). Extracellular glucose influences osteoblast differentiation and c-Jun expression. J Cell Biochem.

[130] Botolin S, McCabe LR (2006). Chronic hyperglycemia modulates osteoblast gene expression through osmotic and non-osmotic pathways. J Cell Biochem.

[131] Cunha JS, Ferreira VM, Maquigussa E, Naves MA, Boim MA (2014). Effects of high glucose and high insulin concentrations on osteoblast function in vitro. Cell Tissue Res.

[132] Liu W, Zhu X, Wang Q, Wang L (2013). Hyperglycemia induces endoplasmic reticulum stress-dependent CHOP expression in osteoblasts. Exp Ther Med.

[133] Pereira RC, Stadmeyer LE, Smith DL, Rydziel S, Canalis E (2007). CCAAT/Enhancer-binding protein homologous protein (CHOP) decreases bone formation and causes osteopenia. Bone.

[134] Lane JC, Butler KL, Poveda-Marina JL, Martinez-Laguna D, Reyes C, de Bont J, Javaid MK, Logue J, Compston JE, Cooper C, Duarte-Salles T, Furniss D, Prieto-Alhambra D (2020). Preschool Obesity Is Associated With an Increased Risk of Childhood Fracture: A Longitudinal Cohort Study of 466,997 Children and Up to 11 Years of Follow-up in Catalonia, Spain. J Bone Miner Res.

[135] Kessler J, Koebnick C, Smith N, Adams A (2013). Childhood obesity is associated with increased risk of most lower extremity fractures. Clin Orthop Relat Res.

[136] Rikkonen T, Sund R, Sirola J, Honkanen R, Poole KES, Kroger H (2021). Obesity is associated with early hip fracture risk in postmenopausal women: a 25-year follow-up. Osteoporos Int.

[137] Jain RK, Vokes T (2022). Fat mass has negative effects on bone, especially in men: a cross-sectional analysis of NHANES 2011–2018. J Clin Endocrinol Metab.

[138] Tencerova M, Figeac F, Ditzel N, Taipaleenmaki H, Nielsen TK, Kassem M (2018). High-fat diet-induced obesity promotes expansion of bone marrow adipose tissue and impairs skeletal stem cell functions in mice. J Bone Miner Res.

[139] Tencerova M, Frost M, Figeac F, Nielsen TK, Ali D, Lauterlein JL, Andersen TL, Haakonsson AK, Rauch A, Madsen JS, Ejersted C, Hojlund K, Kassem M (2019). Obesity-associated hypermetabolism and accelerated senescence of bone marrow stromal stem cells suggest a potential mechanism for bone fragility. Cell Rep.

[140] Tencerova M, Rendina-Ruedy E, Neess D, Faergeman N, Figeac F, Ali D, Danielsen M, Haakonsson A, Rosen CJ, Kassem M (2019). Metabolic programming determines the lineage-differentiation fate of murine bone marrow stromal progenitor cells. Bone Res.

[141] Ulum B, Teker HT, Sarikaya A, Balta G, Kuskonmaz B, Uckan-Cetinkaya D, Aerts-Kaya F (2018). Bone marrow mesenchymal stem cell donors with a high body mass index display elevated endoplasmic reticulum stress and are functionally impaired. J Cell Physiol.

[142] Hein GE (2006). Glycation endproducts in osteoporosis–is there a pathophysiologic importance?. Clin Chim Acta.

[143] Khajuria DK, Soliman M, Elfar JC, Lewis GS, Abraham T, Kamal F, Elbarbary RA (2020). Aberrant structure of fibrillar collagen and elevated levels of advanced glycation end products typify delayed fracture healing in the diet-induced obesity mouse model. Bone.

[144] Ogawa N, Yamaguchi T, Yano S, Yamauchi M, Yamamoto M, Sugimoto T (2007). The combination of high glucose and advanced glycation end-products (AGEs) inhibits the mineralization of osteoblastic MC3T3-E1 cells through glucose-induced increase in the receptor for AGEs. Horm Metab Res.

[145] Rivas A, Vidal RL, Hetz C (2015). Targeting the unfolded protein response for disease intervention. Expert Opin Ther Targets.

[146] Wang Z, Li Z, Wang G, Sun Y, Yuan Y, Nie H (2021). Salubrinal alleviates collagen-induced arthritis through promoting P65 degradation in osteoclastogenesis. Int J Mol Sci.

[147] Kimura F, Miyazawa K, Hamamura K, Tabuchi M, Sato T, Asano Y, Kako S, Aoki Y, Sugita Y, Maeda H, Togari A, Goto S (2021). Suppression of alveolar bone resorption by salubrinal in a mouse model of periodontal disease. Life Sci.

[148] Yokota H, Hamamura K, Chen A, Dodge TR, Tanjung N, Abedinpoor A, Zhang P (2013). Effects of salubrinal on development of osteoclasts and osteoblasts from bone marrow-derived cells. BMC Musculoskelet Disord.

[149] He L, Lee J, Jang JH, Sakchaisri K, Hwang J, Cha-Molstad HJ, Kim KA, Ryoo IJ, Lee HG, Kim SO, Soung NK, Lee KS, Kwon YT, Erikson RL, Ahn JS, Kim BY (2013). Osteoporosis regulation by salubrinal through eIF2alpha mediated differentiation of osteoclast and osteoblast. Cell Signal.

[150] Hamamura K, Nishimura A, Iino T, Takigawa S, Sudo A, Yokota H (2015). Chondroprotective effects of Salubrinal in a mouse model of osteoarthritis. Bone Joint Res.

